# Treatment of COVID-19 Acute Respiratory Distress Syndrome With a Tabletop Noninvasive Ventilation Device in a Respiratory Intermediate Care Unit

**DOI:** 10.1016/j.mayocpiqo.2022.04.001

**Published:** 2022-04-19

**Authors:** Barney Thomas Jesudason Isaac, Nadesan Priya, Avinash Anil Nair, Balamugesh Thangakunam, Amith Balachandran, Tina George, Sheba Meriam Thomas, Tarun Kottukulam George, Ramya Iyadurai, Selwyn Selva Kumar, Anand Zachariah, Bhagteshwar Singh, Priscilla Rupali, Kishore Pichamuthu, Richa Gupta, Jefferson Daniel, Jebin Roger Sasikumar, Sujith Thomas Chandy, Devasahayam Jesudas Christopher

**Affiliations:** a Department of Pulmonary Medicine, Christian Medical College, Vellore, India; b Department of Respiratory Medicine, Christian Medical College, Vellore, India; c Department of General Medicine, Christian Medical College, Vellore, India; d Department of Infectious Diseases, Christian Medical College, Vellore, India; e Department of Medical ICU, Christian Medical College, Vellore, India; f Department of Clinical Infection Microbiology and Immunology, Institute for Infection, Veterinary and Ecological Sciences, University of Liverpool, Liverpool, United Kingdom; g Tropical and Infectious Diseases Unit, Royal Liverpool University Hospital, Liverpool, United Kingdom

**Keywords:** ARDS, acute respiratory distress syndrome, BPAP, bilevel positive airway pressure, CARDS, coronavirus disease 2019 acute respiratory distress syndrome, CPAP, continuous positive airway pressure, COVID-19, coronavirus disease 19, HFNO, high-flow nasal oxygen, ICU, intensive care unit, IMV, invasive mechanical ventilation, IPAP, inspiratory positive airway pressure, NIV, noninvasive ventilation, PEEP, positive end-expiratory pressure, RIMCU, respiratory intermediate care unit

## Abstract

**Objective:**

To study the outcomes of noninvasive ventilation (NIV) administered through a tabletop device for coronavirus disease 2019 acute respiratory distress syndrome in the respiratory intermediate care unit (RIMCU) at a tertiary care hospital in India.

**Patients and Methods:**

We retrospectively studied a cohort of hospitalized patients deteriorating despite low-flow oxygen support who received protocolized management with positive airway pressure using a tabletop NIV device in the RIMCU as a step-up rescue therapy from July 30, 2020 to November 14, 2020. Treatment was commenced on the continuous positive airway pressure mode up to a pressure of 10 cm of H_2_O, and if required, inspiratory pressures were added using the bilevel positive air pressure mode. Success was defined as weaning from NIV and stepping down to the ward, and failure was defined as escalation to the intensive care unit, the need for intubation, or death.

**Results:**

In total, 246 patients were treated in the RIMCU during the study period. Of these, 168 received respiratory support via a tabletop NIV device as a step-up rescue therapy. Their mean age was 54 years, and 83% were men. Diabetes mellitus (78%) and hypertension (44%) were the commonest comorbidities. Treatment was successful with tabletop NIV in 77% (129/168) of the patients; of them, 41% (69/168) received treatment with continuous positive airway pressure alone and 36% (60/168) received additional increased inspiratory pressure via the bilevel positive air pressure mode.

**Conclusion:**

Respiratory support using the tabletop NIV device was an effective and economical treatment for coronavirus disease 2019 acute respiratory distress syndrome. Further studies are required to assess the appropriate time of initiation for maximal benefits and judicious utilization of resources.

Coronavirus disease 19 (COVID-19), caused by severe acute respiratory syndrome coronavirus 2, has ravaged the world, not only on account of the large number of individuals infected but also with regard to mortality, mostly due to respiratory failure. In approximately 81% of symptomatic patients, the disease is mild, and these individuals are likely to get better without any specific measures.^1^ Approximately 14% have moderate symptoms and need hospitalization because of hypoxia but do not need care in the intensive care unit (ICU). However, an additional 5% may require care in the ICU because of refractory hypoxia or multiorgan failure.^1^ The need for invasive mechanical ventilation (IMV) in those who are critically ill is high. The outcomes of IMV were abysmal, as determined in initial reports. In a report from 552 hospitals across China,^2^ the mortality rate of those who received IMV was 60% (assuming that all those who died in the hospitals were on ventilation). This is despite one fifth of the patients receiving extracorporeal membrane oxygenation (ECMO). In 2 subsequent reports from China, the mortality rate among those who received IMV was 86%^3^ and 97%.^4^ Reports from 2 large cohorts from New York^5^ and the United Kingdom^6^ found better outcomes, with a mortality rate of 24.5% and 37%, respectively, in those who received IMV; however, it has to be kept in mind that 72% and 46% of the patients, respectively, remained in the hospital at the time of reporting.

Noninvasive respiratory support strategies are an attractive option to avoid the need for IMV and its inherent risks.^7^ It can be provided either through the high-flow nasal oxygen (HFNO) or noninvasive ventilation (NIV) mode of a ventilator or a tabletop NIV device. Although HFNO consumes high volumes of oxygen, which is both expensive and precious, providing NIV through a ventilator is both expensive and requires high levels of expertise. Noninvasive ventilation has been extensively recommended for the management of respiratory failure, predominantly caused by chronic obstructive pulmonary disease^8^, ^9^, ^10^ and obesity,^11^ both in hospitals and homes because tabletop NIV is a suitable option for both. Because it can provide a positive end-expiratory pressure for the treatment of obstructive sleep apnea and pressure support as required for the treatment of chronic obstructive pulmonary disease or obesity hypoventilation syndrome, its use can be extended beyond to treat other causes of type 1 and 2 respiratory failure. It is often administered outside the ICU in medical and respiratory wards. Respiratory physicians and respiratory therapy teams are conversant with its use. These devices are less expensive, are readily available for purchase in large numbers, and could be deployed for the care of patients with COVID-19. However, there is a risk that this could delay the deployment of IMV and, thus, worsen ultimate outcomes. There is also a fear that the use of these devices could increase the risk of infection among health care workers.^7^ These have resulted in variable practices across the world.

The details of uncertainty about the choice of respiratory support in patients with coronavirus disease 2019 acute respiratory distress syndrome (CARDS) was assessed via a survey of ICU specialists conducted in 85 countries to ascertain the choice of respiratory support using a case vignette of severe hypoxemia due to COVID-19 infection. High-flow nasal oxygen was preferred by 47% of the specialists, continuous positive airway pressure (CPAP) or NIV by 26%, immediate tracheal intubation by 7%, and the rest chose only to optimize conventional oxygen therapy.^12^ Studies from the United Kingdom^13^^,^^14^ found that the early initiation of CPAP is probably beneficial and could possibly decrease the need for IMV and decrease mortality. There are reports from Italy^15^ of the use of a successful exclusive NIV service as a bridge between care in wards and ICUs. We report herewith our experience with the early use of a tabletop NIV device in an intermediate care ward, exclusively set up for this purpose.

## Patients and Methods

This was a retrospective study of consecutive patients with CARDS referred for treatment with NIV to the respiratory intermediate care unit (RIMCU) of our tertiary care referral hospital in southern India from July 30, 2020 to November 14, 2020. The study was approved by the institutional review board or ethics committee (IRB minute no. 13612, dated November 25, 2020).

The first wave of COVID-19 in India commenced in March 2020, and as the number of cases surged by July 2020, our hospital resources were stretched. There was a woeful shortage of beds for critical care. The respiratory medical ward was converted into a 14-bedded RIMCU to address the need. Each bed was provided with a multiparameter monitor capable of continuously monitoring oxygen saturation, heart rate, and respiratory rate. The RIMCU was air-conditioned, and negative pressure was set up to ensure 12 air exchanges per hour to improve the safety of health care workers. The ratio of the number of patients to that of respiratory therapists, nurses, and doctors was 1:5, 1:3, and 1:5, respectively.

The service was designed for patients with reverse transcription polymerase chain reaction-proven COVID-19 with a worsening respiratory status from COVID-19 wards and the emergency department. They were accepted if they fulfilled the following criteria: (1) an oxygen requirement of more than 40% through a venturi mask or more than 5 L/min through nasal prongs to maintain a target SpO_2_ of 92% or more and/or (2) increased work of breathing, as indicated by a respiratory rate of more than 30 breaths/minute and the use of accessory muscles for respiration. Patients who required more than 60% of the fraction of inspired oxygen (FiO_2_) to maintain the target saturation level, those with impending respiratory arrest, and those with absolute contraindications for NIV were not received into the RIMCU and were admitted directly to the ICU. A few patients who received NIV were stepped down from the ICU for weaning before being shifted to the medical ward. They were not included in the analysis.

With a view to prioritize the available resources for those in whom maximum benefits were likely, escalation plans were drawn out at admission as per the criteria formulated by the COVID-19 clinical treatment group, in consultation with the patients and their family. This was performed on the basis of age, comorbidities and functional status prior to current illness which are likely to influence the outcomes:

(1) “For full escalation” implied that the plan was to shift the patient to the ICU if the patient’s health deteriorates despite NIV, fulfilling predefined criteria for transfer to the ICU.
(2) “NIV as the ceiling of care” implied that the patient would not be transferred to the ICU even if they fulfilled the criteria for transfer to the ICU, and management would continue in the RIMCU till the patient improved or died.

### Tabletop NIV Treatment

A chest x-ray was performed before the initiation of NIV to perform a baseline assessment of lung infiltrates and rule out the possibility of pneumothorax, which is a contraindication for NIV.

**Figure 1:**
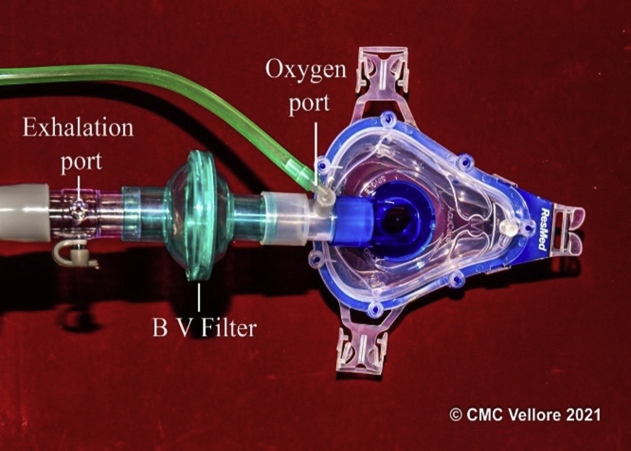
Circuit with a mask and connections.

Positive airway pressure was administered using 1 of the following tabletop NIV devices: A40 (Philips) or Stellar 150 or Stellar 100 (ResMed). The outflow from the device was delivered to the patient through a single-limb circuit and a nonvented oronasal mask. Oxygen from the wall (maximum flow, 15 L/minute) through a flow meter was entrained into the circuit closer to the mask (Figure 1), and exhalation was possible through an exhalation port in the circuit, which was placed beyond a bacterial-viral filter, to decrease the viral load in the expired air expelled from the circuit.

**Figure 2:**
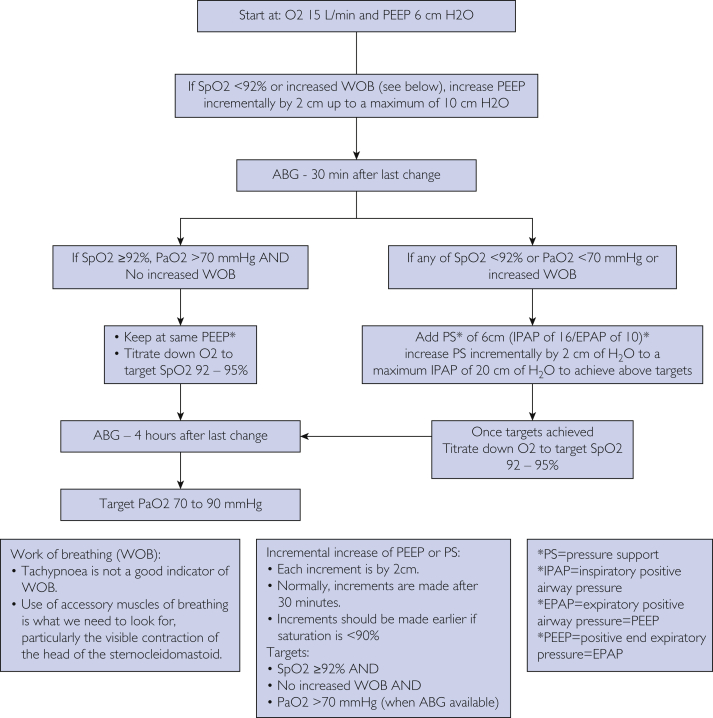
Treatment algorithm followed in the respiratory intermediate care unit using a tabletop noninvasive ventilation device. ABG, arterial blood gas; EPAP, expiratory positive airway pressure; IPAP, inspiratory positive airway pressure; NIV, noninvasive ventilation; PaO_2_, partial pressure of oxygen; PEEP, positive end-expiratory pressure; PS = pressure support; RIMCU, respiratory intermediate care unit; WOB = work of breathing.

The treatment algorithm followed for NIV is outlined in Figure 2. Treatment for all eligible patients was initiated using the CPAP mode at a positive end-expiratory pressure (PEEP) of 6 cm of H_2_O, with an increase in the pressure by 2 cm of H_2_O if the targets were not met. Once a PEEP of 10 cm of H_2_O was reached and the targets were still not met, we switched to the bilevel positive airway pressure (BPAP) mode, and the inspiratory positive airway pressure (IPAP) was increased gradually by 2 cm of H_2_O till the targets were met or till a pressure of 20 cm of H_2_O was reached. The IPAP minus the expiratory positive airway pressure yielded the additional pressure support given during inspiration. Low-dose sedation with dexmedetomidine was used in some patients who had difficulty tolerating NIV. Awake self-positioning was advocated in all patients, with a 2-hour position change calendar, assisted by the nurses and respiratory therapists.

### Estimating Delivered FiO_2_

Although oxygen can be delivered through the circuit of these tabletop NIV devices, because of the absence of an oxygen blender, the delivered FiO_2_ is unknown. Therefore, we conducted experiments using a test lung and an FiO_2_ meter, which resulted in the creation of an FiO_2_ chart (Table 1). We used this to estimate delivered FiO_2_, thus facilitating the titration of the administration of oxygen, and the oxygen saturation to the fraction of inspired oxygen (SpO_2_/FiO_2_) and partial pressure of oxygen to the fraction of inspired oxygen (PaO_2_/FiO_2_) ratios were calculated.

**Table 1:** FiO_2_ Reference Chart for Tabletop NIV Device With Entrained Oxygen^a^

BPAP/CPAP		Oxygen flow in L/min entrained							
		0	2	4	6	8	10	12	15
IPAP (cm of H_2_O)	EPAP (cm of H_2_O)	Measured FiO_2_ in %^b^							
-	6	21	30	38	52	59	71	85	97
-	8	21	27	35	46	47	62	65	80
-	10	21	26	32	40	46	50	55	67
-	12	21	25	32	37	42	46	48	58
16	10	21	26	31	37	42	48	52	61
18	10	21	26	30	34	39	44	48	57
20	10	21	25	29	33	37	42	46	54

a BPAP, bilevel positive airway pressure; CPAP, continuous positive airway pressure; EPAP, expiratory positive airway pressure; FiO_2_, fraction of inspired oxygen; IPAP, inspiratory positive airway pressure.

b All measurements performed with oxygen entry closer to the mask and away from the exhalation port as seen in Figure 1. IPAP − EPAP = pressure support.

### Weaning Algorithm

Once stabilized, the pressures were progressively reduced; if on the BPAP mode, a switch was made to the CPAP mode, and if stable for at least 24 hours, protocolized weaning was initiated: 3:1 (3 hours on NIV and 1 hour on oxygen via a nasal cannula or face mask), followed by 2:2 and 1:3 and then oxygen alone. Once stable for 24 hours on oxygen alone, the patient was shifted to the ward.

### Criteria for Stepping Up to ICU

For those planned for full escalation, the indications for step-up to the ICU were as follows:

(1) Failure to meet the saturation targets or persistent increased work of breathing despite a maximum IPAP of 20 cm of H_2_O, maximum expiratory positive airway pressure of 10 cm of H_2_O, and up to 15 L/minute of oxygen entrained through the device.
(2) Drop in the Glasgow Coma Scale score to below 8/15.
(3) Clinical deterioration and not tolerating the tabletop NIV device.

### Success and Failure

Successful weaning from NIV and stepping down to the ward was considered as success with tabletop NIV. Transfer to the ICU or intubation or /death in the RIMCU was considered as failure.

### Data Extraction and Analysis

Patient data were extracted from their electronic medical records and monitoring sheets maintained in the RIMCU. The data were analyzed using SPSS, version 21 (License number (customer ID): 200699; Vendor: SPSS South Asia Pvt Ltd). Continuous variables were expressed as mean and SD or as median and interquartile range. Categorical variables were expressed as frequencies and percentages.

## Results

A total of 246 patients with CARDS were managed in the RIMCU during the study period. Of these, 168 fulfilled the criteria to be included in the analysis and 78, who were stepped down from the ICU for weaning, were excluded. The clinicodemographic details of the patients are described in Table 2. Diabetes mellitus (78%) and hypertension (44%) were the commonest comorbidities. There was a low prevalence of respiratory comorbidities (17.8%). On admission to the RIMCU, the mean partial pressure of oxygen to the fraction of inspired oxygen ratio, oxygen saturation to the fraction of inspired oxygen ratio, modified Sequential Organ Failure Assessment, quick COVID19 Severity Index, quick Sequential Organ Failure Assessment, and FiO_2_ of these patients was 211, 212, 2.5, 7.1, 0.99, and 51%, respectively. The mean duration of the use of NIV in the RIMCU was 6 days.

**Table 2:** Characteristics of the Patients on Admission to the Hospital

Variables	Mean (SD)/median (IQR)/frequency (%) (n=168)
Age in years	53.6 (11.7)
Sex	
Male	140 (83.3%)
Female	28 (16.7%)
Body mass index in kg/m^2^ (n=79)	27.7(5.8)
Shifted from	
From COVID-19 wards	162 (96.4%)
From emergency department	6 (3.6%)
Symptoms at presentation	
Asymptomatic	1 (0.6%)
Cough	131 (78%)
Shortness of breath	144 (85.7%)
Fever	145 (86.3%)
Fatigue	28 (16.7%)
Myalgia	31 (18.5%)
Diarrhea	7 (4.2%)
Sore throat	8 (4.8%)
Smell and taste disturbances	4 (2.4%)
Comorbidities	
Diabetes mellitus	131 (78%)
Hypertension	73 (43.5%)
Ischemic heart disease	12 (7.1%)
Congestive cardiac failure	6 (3.6%)
Dementia	1 (0.6%)
Chronic respiratory diseases	30 (17.8%)
Asthma	14 (8.3%)
COPD	12 (7.1%)
ILD	4 (2.4%)
Blood parameters	
C-reactive protein (n=23)	71.5 (50.6)
D-dimer (n=152)	644 (439-991)
Ferritin (n=143)	630.7 (565.7)
N/L ratio (n=151)	8.7 (6.9)
LDH (n=131)	806.8 (233.6)
WHO severity	
Mild	13 (7.7%)
Moderate	48 (28.6%)
Severe	95 (56.5%)
Critical	12 (7.1%)

COPD, chronic obstructive pulmonary disease; COVID-19, coronavirus disease 2019; ILD, interstitial lung disease; IQR, interquartile range; LDH, lactose dehydrogenase; N/L, neutrophil/lymphocyte; WHO, World Health Organization.

**Figure 3:**
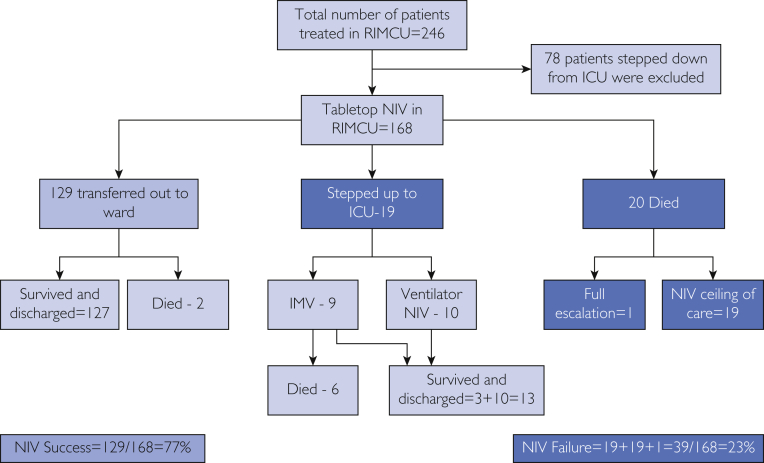
Outcome of patients in the respiratory intermediate care unit. ICU, intensive care unit; IMV, invasive mechanical ventilation; NIV, noninvasive ventilation; RIMCU, respiratory intermediate care unit.

Figure 3 depicts the outcomes in patients in whom treatment with tabletop NIV was initiated. Of the 168 patients who were stepped up for the initiation of tabletop NIV, 129 (77%) had a successful outcome. Among those in whom tabletop NIV failed, 20 died in the RIMCU and 19 were stepped up to the ICU because they fulfilled the criteria for the same. Of the 19 patients who were stepped up to the ICU, 9 needed mechanical ventilation, of whom 3 improved and 6 died. The other 10 patients could be successfully managed without intubation, with higher oxygen concentrations delivered via the “NIV mode” of the mechanical ventilator. Among the 20 patients who died in the RIMCU, only 1 patient was considered for full escalation; the other 19 patients were considered for “NIV as the ceiling of care” and, hence, were not stepped up to the ICU. During treatment with NIV in the RIMCU, pneumothorax developed in only 1 patient, which was successfully managed with an intercostal chest drain.

**Figure 4:**
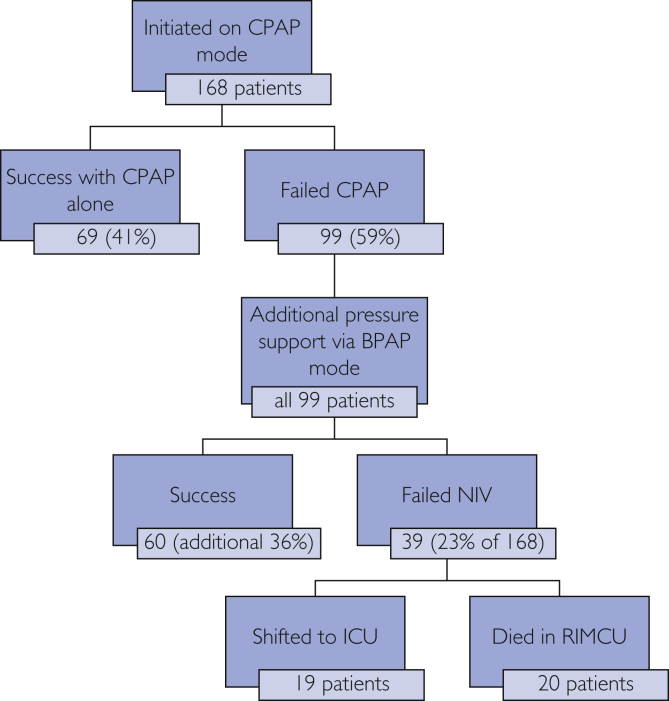
Outcomes with continuous positive airway pressure and bilevel positive airway pressure modes of treatment. BPAP, bilevel positive airway pressure; CPAP, continuous positive airway pressure; ICU, intensive care unit; NIV, noninvasive ventilation; RIMCU, respiratory intermediate care unit.

In the tabletop NIV device, because our algorithm involved the initiation of treatment using the CPAP mode initially and, only if required, stepping up to receive higher inspiratory pressures via the BPAP mode, we were able to assess the success of these modes (Figure 4). A total of 69 patients were successfully weaned while on the CPAP mode alone; this amounted to a success rate of 41% among the patients. The 99 patients who could not be successfully treated with maximal CPAP pressures went on to receive additional pressure support (via the BPAP mode), and 61 (62%) of these patients had a successful outcome. Thus, the additional success rate with higher inspiratory pressures via the BPAP mode was 36% (Figure 4).

## Discussion

When we faced a surge of patients hospitalized for oxygen therapy in the COVID-19 medical wards, some of whom worsened, requiring higher respiratory support, the capacity of our ICUs was overwhelmed. This prompted us to consider setting up the RIMCU to use tabletop NIV as a rescue therapy to avoid care in the ICU and mechanical ventilation. A systematic review and network meta-analysis^16^ of acute hypoxemic respiratory failure, published in 2020, concluded that noninvasive oxygenation strategies, compared with standard oxygen therapy, was associated with a lower risk of death and lesser need for IMV. However, the studies included in this meta-analysis were predominantly on community-acquired pneumonia. In the pre-COVID-19 era, the European Respiratory Society/American Thoracic Society clinical practice guidelines^10^ published in 2017 cautiously recommended NIV for patients with acute hypoxemic respiratory failure secondary to community-acquired pneumonia or early acute respiratory distress syndrome (ARDS). It has been proposed as a preventive strategy for avoiding intubation only in a subset of highly selected co-operative patients when it is performed by an experienced team in the absence of any major organ dysfunction. It is known that lungs with ARDS are stiff, often called as “baby lungs” with poor compliance,^17^ often requiring IMV with strategies involving high PEEP, low tidal volumes, permissive hypercapnia, recruitment maneuvers, and prone positioning. However, in the earlier stage of COVID-19, the lung appears to be compliant with low elastance, described as L-type,^17^ and, hence, can generate good volumes. Therefore, it is likely to be amenable to NIV. If it progresses, then compliance decreases in later stages, with high elastance, called as H-type^17^ ARDS, which behaves like conventional non-CARDS and is more likely to require IMV.

In our cohort, the success rate of the use of the tabletop NIV device in patients with CARDS was 77% (129 out of 168). This is higher than the rate in most published studies that have used CPAP or BPAP in patients with COVID-19, which have reported success rates ranging from 33% to 70%.^18^, ^19^, ^20^, ^21^, ^22^, ^23^, ^24^ We believe that the following 4 factors could have played a role in the good success rate in our cohort: the timely initiation of NIV, a dedicated NIV ward (RIMCU) and skilled workforce experienced in administering NIV treatment, the addition of pressure support beyond just CPAP, and the use of awake self-proning.

Although the role of NIV in the management of CARDS is becoming increasingly recognized, the time of initiation and the mode of delivery are yet to be established. Our experience is consistent with the advantage of early initiation of CPAP, as reported by researchers from the United Kingdom.^13^^,^^14^ We hypothesized that the early initiation of respiratory support could prevent patient self-inflicted lung injury, which is initiated and propagated by massive unsupported inspiratory efforts by these patients. This has been previously proposed^25^ for spontaneously breathing patients with CARDS. A recent simulation study^26^ with mathematical computation reported that patient self-inflicted lung injury is comparable with ventilator-induced lung injury. Hence, we used the requirement of either supplemental oxygen or the work of breathing to select suitable patients.

The setting up of a dedicated area (RIMCU), with adequate monitoring devices, and the deployment of adequately skilled workforce—doctors, nurses, and respiratory therapists—round the clock was key to our success. The success of similar models has been reported by Radovanovic et al,^15^ Bellani et al,^20^ and Coppadoro et al^21^ from Italy.

Because COVID-19 results in hypoxemic respiratory failure, most experts believe that CPAP or PEEP alone is beneficial because it helps in keeping the alveoli open during expiration and enhances oxygenation; this has resulted in most NIV trials in patients with COVID-19 using only CPAP.^18^, ^19^, ^20^, ^21^, ^22^, ^23^, ^24^^,^^27^^,^^28^ However, another aspect of CARDS is increased work of breathing. Although it is true that the splinting effect of PEEP can partly take care of this, we hypothesized that adding pressure support may have a role in those in whom CPAP fails. In our cohort, the success rate of the CPAP mode alone was 41%, which is comparable with the rates in some of the other reports that have used the CPAP mode alone.^18^, ^19^, ^20^, ^21^, ^22^, ^23^, ^24^ However, by adding pressure support via the BPAP mode, our success rate increased by 36%, resulting in an overall success rate of 77% with tabletop NIV. Thus, our study has demonstrated the merit of this strategy.

We would like to propose the following explanation for the benefit of adding pressure support via the BPAP mode over the CPAP mode in those in whom the latter fails. Adding pressure support decreases the work of breathing. It also increases minute ventilation, which in turn increases alveolar ventilation when the respiratory rate is not further increased (minute ventilation = tidal volume × respiratory rate; alveolar ventilation = tidal volume − anatomic dead space). The increase in alveolar ventilation would result in an increase in oxygenation. On the contrary, higher the pressure, lower will be the FiO_2_ for the same flow of oxygen (Table 1). When an increase in the pressure does not result in an increase in the tidal volume, there is an increase in the risk of barotrauma, without an increase in oxygenation. Similarly, it is known that an increase in the tidal volume beyond 6 mL/kg in patients with ARDS increases the risk of volutrauma. However, we had only 1 occurrence of pneumothorax in our cohort despite all of them receiving CPAP or BPAP. We had a strict ceiling PEEP of 10 cm of H_2_O and IPAP of 20 cm of H_2_O; this could have been a reason for the low rates of barotrauma.

Our study has a few limitations. First, the retrospective nature of the study has its own inherent issues with the completeness of data; however, each patient in the RIMCU had documentation sheets that captured most of the required information. The second limitation is the narrow oxygen requirement window (40%-60% FiO_2_) in which the intervention was used, but this range is important. Patients requiring less than 40% FiO_2_ in the absence of increased work of breathing are likely to improve without positive pressure. Tabletop NIV cannot deliver an FiO_2_ of more than 60%; additionally, all patients who worsened and required invasive ventilator support would have gone through this window. Third, a major limitation of our study is the absence of a control arm in which oxygen therapy was continued till invasive ventilation was required. Lawton et al^14^ compared the outcomes of their CPAP cohort from the United Kingdom with a large national cohort in the United Kingdom and found that the use of CPAP decreased admissions for critical care and the need for invasive ventilators by at least half. Therefore, there could be ethical issues in performing a study comparing NIV with oxygen therapy alone. Lastly, the mean body mass index (calculated as the weight in kilograms divided by the height in meters squared) in our cohort was 27.7 kg/m^2^; hence, the applicability of the success rates to cohorts with higher body mass indexes needs evaluation.

## Conclusion

Noninvasive ventilation through a tabletop device is a safe, effective, and affordable treatment for CARDS and is likely to prevent the need for care in the ICU, need for invasive ventilation, and, possibly, mortality in hospitalized patients deteriorating despite low-flow oxygen support. An exclusive setup with adequate monitoring may be essential for optimal outcomes. Further studies are required to confirm the appropriate time for the initiation of NIV for maximal benefit and judicious resource utilization.

## Potential competing interests

The authors report no competing interests.
